# Lipopolysaccharide detection by the innate immune system may be an uncommon defence strategy used in nature

**DOI:** 10.1098/rsob.220146

**Published:** 2022-10-05

**Authors:** Anna E. Gauthier, Randi D. Rotjan, Jonathan C. Kagan

**Affiliations:** ^1^ Division of Gastroenterology, Boston Children's Hospital and Harvard Medical School, 300 Longwood Avenue, Boston, MA 02115, USA; ^2^ Program in Virology, Harvard Medical School, Boston, MA, USA; ^3^ Department of Biology, Boston University, 5 Cummington Mall, Boston, MA 02215, USA; ^4^ Harvard Medical School, and Boston Children's Hospital, Division of Immunology, Division of Gastroenterology, USA

**Keywords:** LPS, TLR4, innate immunity, inflammasomes, inflammation, pattern recognition

## Abstract

Since the publication of the Janeway's Pattern Recognition hypothesis in 1989, study of pathogen-associated molecular patterns (PAMPs) and their immuno-stimulatory activities has accelerated. Most studies in this area have been conducted in model organisms, which leaves many open questions about the universality of PAMP biology across living systems. Mammals have evolved multiple proteins that operate as receptors for the PAMP lipopolysaccharide (LPS) from Gram-negative bacteria, but LPS is not immuno-stimulatory in all eukaryotes. In this review, we examine the history of LPS as a PAMP in mammals, recent data on LPS structure and its ability to activate mammalian innate immune receptors, and how these activities compare across commonly studied eukaryotes. We discuss why LPS may have evolved to be immuno-stimulatory in some eukaryotes but not others and propose two hypotheses about the evolution of PAMP structure based on the ecology and environmental context of the organism in question. Understanding PAMP structures and stimulatory mechanisms across multi-cellular life will provide insights into the evolutionary origins of innate immunity and may lead to the discovery of new PAMP variations of scientific and therapeutic interest.

## Graphical abstract

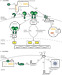

## 1. Introduction

The ability to distinguish self from non-self is fundamental to the survival of all eukaryotic life. At a molecular level, this is accomplished by pattern recognition receptors (PRRs) of eukaryotic cells that recognize conserved molecules of microorganisms that are distinctly non-self [1]. These molecules come in the form of microbial cell wall components and nucleic acids, and are termed pathogen-associated molecular patterns (PAMPs). Examples of PAMPs include the lipid A subregion of bacterial lipopolysaccharide (LPS), the flagellin subunit of bacterial flagella as well as microbial RNA and DNA in association with individual nucleotides [2]. Based on the pattern recognition model, originally posited by Charles Janeway Jr in 1989 [3], it has been assumed that PRRs of eukaryotes should have the ability to detect all members of a given class of microorganisms via their conserved PAMPs. The only exception to this rule should be microorganisms with altered PAMP structures that prevent PRR detection, which may result from coevolution with the host. Therefore, apart from pathogens or beneficial commensals that have an evolutionary history with a eukaryotic host, PRRs should have the ability to detect all microorganisms [2,4]. The assumption of near-universal PAMP detection by PRRs is a foundation of modern immunology that has been tested many times since Janeway published this theory. Nevertheless, it remains to be determined (i) whether PRRs of eukaryotes detect all microorganisms that have not co-evolved with the host, and (ii) whether PAMPs evolved to be universally detected by eukaryotes spanning the evolutionary tree. In this review, we use LPS as a case study for investigating the universality of PAMP detection. In mammals (humans and mice), LPS is one of the most well-studied PAMPs, making it a strong candidate for evaluation and comparison to other known PAMPs. The goal of this review is to summarize what is known about LPS to date, and to highlight avenues for further investigation of this microbial product and others.

## 2. Discovery of lipopolysaccharide lipid A and its role as a pathogen-associated molecular pattern in innate immunity

Humans have been studying LPS and its mechanism of action on the mammalian immune system for over 150 years. One of the first rudimentary isolations of LPS was probably made in 1856 along with the discovery of its ability to cause disease. At the time, it was referred to as ‘putrid poison’ and was implicated experimentally in the onset of fever and lethal septic shock in dogs by the Danish physiologist and medical doctor, Peter Ludvig Panum. Panum incubated raw meat in water at room temperature until it smelled ‘putrid’, at which time the substance was passed through a series of filters until macroscopically free of bacterial particles. Injection of dogs with the substance led to disease (fever, vomiting and vascular collapse), followed by either rapid death or slow recovery, depending on the animal and dose. Panum hypothesized that this substance was responsible for the symptoms of sepsis he observed in his patients [5,6].

Over 30 years later in 1892, ‘putrid poison’ was further purified and dubbed endotoxin by Richard Pfeiffer, a student of Robert Koch [7]. Pfeiffer hypothesized that disease caused by *Vibrio cholera* was not dependent on bacterial viability, but on a toxic compound (endotoxin) from the bacteria. An experiment performed with heat-killed *V. cholera* demonstrated that the toxic substance could withstand prolonged heating at 100°C and was located in the bacterial cell wall [8]. Andre Boivin, Ion Mesrobeanu and Lydia Mesrobeanu were the first group of scientists to observe that endotoxin contained a polysaccharide region and a lipid region, a molecular moiety that would come to be known as Gram-negative bacterial LPS [9–11]. In 1952, Dr Otto Westphal and Dr Otto Lüderitz developed the hot phenol method still used to purify LPS today (though it has been since modified) [12], and in 1954, the method to precipitate the lipid region from LPS [13]. Westphal and Lüderitz proposed that this region (named lipid A) was responsible for the toxic activities of LPS [14]. Lipid A was confirmed to be the toxic region of LPS by Dr Ernst Rietschel, Westphal and Lüderitz in 1971, when they determined that the polysaccharide regions of LPS were not required for the lipid A region's observed toxicity [15]. In 1975, Dr Chris Galanos directly demonstrated that solubilized lipid A was responsible for the endotoxic activities of LPS [16]. In 1982, Rietschel's laboratory solved the structure of lipid A from *Salmonella minnesota* [17], and in 1983, the structures of lipid A from *E. coli* [18] and *Salmonella enterica* serovar Typhimurium [19] were solved.

While pathologic sepsis-inducing activities of LPS were used to identify and characterize the activities of this PAMP experimentally, it is now appreciated that the detection of LPS by select PRRs initiates beneficial defensive responses to infection [20]. Toll-like receptor 4 (TLR4) is one of the first identified members of the PRR superfamily present in mammals. This protein was identified as a likely PRR by Medzhitov, Janeway and colleagues in 1997, as they found human TLR4 (first known as hToll) to be homologous to the *Drosophila melanogaster* Toll protein [21], which had been previously identified as a sensor of fungal infections by Lemaitre *et al*. [22]. When constitutively activated, human TLR4 was found to activate nuclear factor kappa B (NF-κB) and upregulate several activities necessary for adaptive immunity [21]. Medzhitov & Janeway [23] hypothesized that this human TLR4 protein functioned to detect PAMPs. Studies in mice provided the first genetic evidence to support this prediction. Decades prior to the work of Medzhitov and Janeway, two groups separately discovered mouse strains (C3H/HeJ and C57BL/10ScCr) that could not recognize LPS and displayed increased host susceptibility to Gram-negative bacterial infections [24–26]. A year after the work of Medzhitov and Janeway, Poltorak *et al*. [27] identified mutations in the TLR4 gene in these strains of mice that rendered TLR4 incapable of recognizing LPS; thus, directly linking LPS recognition in mice to TLR4. This finding was validated by several near-contemporaneous studies [28,29].

In addition to TLR4, several other mammalian proteins have been identified as PRRs that detect LPS. These include lipopolysaccharide-binding protein (LBP), cluster of differentiation 14 (CD14), myeloid differentiation factor-2 (MD2), brain-specific angiogenesis inhibitor 1 (BAI1), guanylate-binding protein 1 (GBP1) and members of the inflammatory caspase family (caspase-4, caspase-5 and caspase-11). Below we describe the mechanisms of LPS recognition by these distinct PRRs and the consequences of this recognition on inflammation and immunity.

## 3. Importance of lipopolysaccharide to Gram-negative bacteria and mammalian innate immunity to lipopolysaccharide

The LPS molecule is an amphipathic glycolipid that accounts for the majority of molecules present in the outer leaflet of the Gram-negative bacterial outer membrane (OM) [30,31]. As such, it plays a vital role in optimal bacterial cell functions [32]. LPS maintains the integrity of the bacterial OM, providing a tightly regulated permeability barrier that is resistant to environmental assaults such as toxins, detergents, antibiotics and other antimicrobial compounds [33–36]. LPS is thought to be essential to the fitness and function of this class of bacteria. However, there have been three species of Gram-negative bacteria identified that are viable without LPS: *Neisseria meningitidis* [37], *Moraxella catarrhalis* [38] and *Acinetobacter baumannii* [39], which are all pathogens of the mammalian respiratory system. Furthermore, studies which have knocked out essential enzymes required for LPS biosynthesis demonstrate that some bacteria can survive without LPS, at least in the laboratory setting [40]. These examples, although notable, will not be the focus of this review; others have published informative reviews on this topic [36,41,42]. Apart from the examples listed above, Gram-negative bacteria require LPS for viability and optimal fitness [43–46].

The human and murine innate immune systems have evolved multiple PRRs that detect LPS [20]. Upon the detection of prototypical *E. coli* LPS, the mammalian (human/mouse) innate immune system mounts a robust transcription-based inflammatory response [20,47]. This response is dictated by the PRR binding the lipid A region of LPS and the localization of said PRR in the cell; whether it is membrane bound or cytosolic. Membrane-localized PRRs that detect the lipid A region induce both the cytokine (NF-κB-activated) [27,48–50] and interferon (interferon regulatory transcription factor 3 (IRF3)-activated) [51] arms of the innate immune system, whereas cytosolic PRRs induce pore formation in the cell membrane and the cleavage (activation) and release of IL-1 family inflammatory cytokines into the extracellular space [52,53].

**Figure 1.**
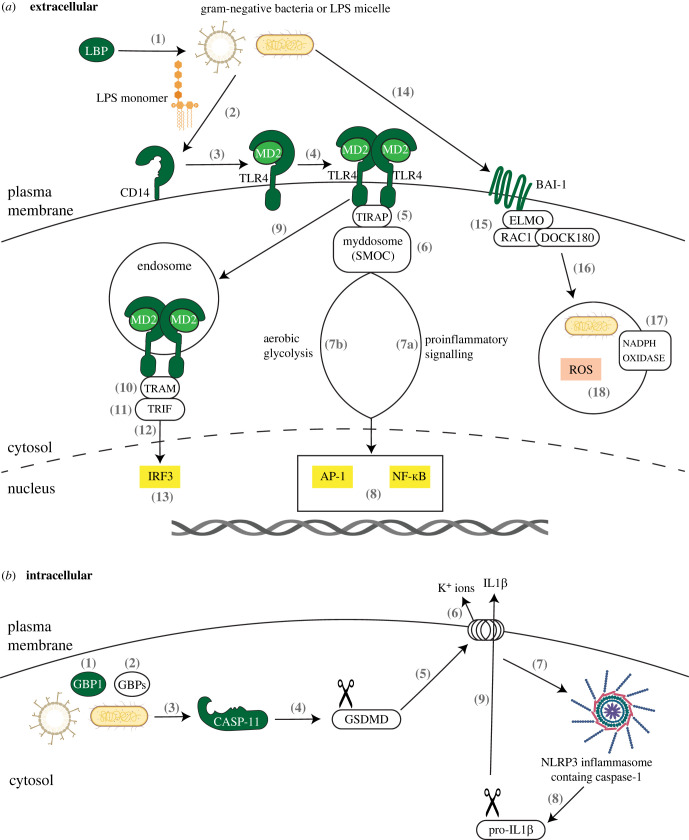
(*Caption overleaf*.) Mammalian PRRs that recognize LPS and the downstream signalling pathways initiated after PAMP recognition. (*a*) Extracellular innate immune pathways are induced following PRR recognition of LPS. (1) LBP interacts with bacterial cell membrane or LPS micelles so that (2) CD14 can extract a monomer of LPS and (3) deliver it to a monomer of MD2/TLR4. (4) The engagement of LPS with the monomer of MD2/TLR4 cross-links this monomer with another monomer of MD2/TLR4, creating a dimer. (5) TIRAP is recruited to the dimer which subsequently results in (6) the formation of the myddosome that (7a) mediates downstream proinflammatory signalling and (7b) the induction of aerobic glycolysis, which (8) culminates in the translocation of transcription factors NF-κB and AP-1 to the nucleus to induce the production of proinflammatory cytokines and mediate metabolic reprogramming. (9) MD2/TLR4 are next endocytosed and TIRAP is replaced by the adaptor proteins (10) TRAM and (11) TRIF, which leads to (12) downstream signalling events in the cytosol that (13) culminate in the translocation of the transcription factor IRF3 to the nucleus and the production of interferon and ISGs. (14) Independent of TLR4, the BAI1 receptor, with seven transmembrane domains, binds to the polysaccharide region of LPS on Gram-negative bacteria and subsequently (15) interacts with ELMO, the DOCK180 complex, and RAC1 to (16) initiate phagocytosis. (17) Phagocytosis activates NADPH-oxidase and (18) the production of ROS. ROS mediates bacterial killing within the phagosome/phagolysosome as a means to host defence. (*b*) Intracellular innate immune pathway induced following Caspase-11 (-4, -5) recognition of LPS. (1) GBP1 associates with the bacterial OM or micelle and (2) recruits other GBPs to the site of interaction. (3) Caspase-11 (-4, -5) associates with GBPs and recognizes LPS from Gram-negative bacteria in the cell cytosol and subsequently (4) cleaves GSDMD. (5) The N-terminal subunit of GSDMD aggregates at the plasma membrane to form pores and (6) potassium ions (K+) are released to the extracellular space, which in turn (7) activates the NLRP3 inflammasome containing caspase-1. (8) Inflammasome-mediated cleavages of pro-IL-1*β* causes (9) active IL-1*β* to be secreted with pores formed by GSDMD. IL-1*β* is now activated and triggers proinflammatory signalling in neighbouring cells and informs the adaptive immune response.

Human and murine phagocytic cells, such as macrophages and dendritic cells, express two main PRR groups that recognize the lipid A region of LPS. TLR4, along with its accessory proteins LBP, CD14 and MD2, functions to detect LPS that is present in the extracellular space (figure 1*a*, 1–13) [54]. LBP circulates in the serum and loosens LPS from the bacterial cell membrane or from LPS micelles [55,56] to enable the extraction of an LPS monomer by CD14 [57–59]. CD14 comes in two forms: a GPI-anchored membrane-bound form and a soluble form that lacks a GPI anchor, both of which bind to LPS monomers [58,60]. CD14 subsequently releases LPS to a complex, composed of MD2 [61] and TLR4 [62,63]. Engagement of MD2 and TLR4 with LPS induces the dimerization with another TLR4-MD2 complex. The acyl chains of the lipid A subregion of LPS cross-link these molecules, leading to dimerization of the cytosolic tails of TLR4, which contain a signalling motif known as a Toll/interleukin-1 receptor (IL-1R) (TIR) domain [64,65]. The dimerized TIR domains are detected by an intracellular protein known as the Toll/interleukin-1 receptor domain-containing adapter protein (TIRAP), which serves as a general sensor for most activated (i.e. dimerized) TLRs [66]. TIRAP interacts with acidic phosphoinositides present in the cytoplasmic leaflet of the plasma and endosomal membranes, thereby surveying these organelles for the presence of dimerized TLRs. TIRAP-mediated TLR detection is achieved through interactions between its TIR domain and those of the upstream receptors. These interactions lead to the assembly of a micron-sized filamentous organelle known as the myddosome, which represents the principal subcellular site of TLR-mediated inflammatory enzyme activation [67]. The myddosome was first defined in cell-free systems with recombinant proteins [68,69] and identified as an endogenous protein complex that is assembled upon TLR activation in macrophages [67]. This complex is a prototypical example of an increasing set of filamentous signalling organelles of the innate immune system, which are collectively known as supramolecular organizing centres (SMOCs) [70,71]. In the case of the myddosome, its' assembly coincides with the activation of the interleukin-1 receptor (IL-1R) associated kinase (IRAK) family kinases within this structure [68]. Activation of the IRAKs initiates a kinase and ubiquitin ligase-dependent cascade that culminates in the induction of aerobic glycolysis [70] and the translocation of the transcription factors activator protein 1 (AP-1), NF-κB (and others) to the nucleus and the subsequent expression of proinflammatory cytokines [72,73].

Within minutes of LPS binding at the plasma membrane, TLR4 is endocytosed in a CD14- and MD2-dependent manner [74]. Within endosomes, the proteins TRIF-related adaptor molecule (TRAM) and TIR domain-containing adaptor-inducing IFN-β (TRIF) are thought to be recruited to the TIRs of TLR4 [75]. How TRAM-TRIF recruitment is coordinated with TIRAP-MYD88 recruitment to the TLR4 TIRs is unknown. Despite this lack of mechanistic insight, it is clear that TRAM-TRIF recruitment stimulates transcription factor activation, with a notable factor being IRF3. In the nucleus, IRF3 induces the expression of interferons and hundreds of interferon-stimulated genes (ISGs) that play diverse roles in host defence [76]. Just as myddosome assembly at the plasma membrane is dependent on MYD88, SMOC formation at the endosomal membrane is likely to be dependent on TRIF, and this complex is referred to as the triffosome [77]. In support of the model that TRIF mediates SMOC formation at the endosomal membrane, multiple proteins have been identified that are recruited to the triffosome in the context of extracellular LPS stimulation and subsequent inhibition of TGFβ-activated kinase (TAK1). Specifically, TRIF binds to the ISG-encoded protein, Z-DNA-binding protein 1 (ZBP1). ZBP1 in turn binds to receptor-interacting serine/threonine-protein kinase 1 (RIPK1) to recruit fas associated via death domain protein (FADD) and caspase-8 to induce cell death and the production and secretion of active IL-1*β* [78]. More recently, extracellular LPS stimulation alone was also demonstrated to result in the formation of the trifosome containing ZBP1 bound to RIPK1. ZBP1 recruitment of RIPK1 was required for the timely interaction of multiple proteins with the triffosome, including TAK1, NF-kappa-B-essential modulator, tumour necrosis factor (TNF) receptor-associated factor 3 (TRAF3) and TANK-binding kinase 1 (TBK1), to drive NF-κB and IRF3-mediated production of proinflammatory cytokines and interferon [79].

LPS is also detected in the cytosolic space of human and murine cells. Human caspase-4 and -5, and murine caspase-11 detect LPS in the host cell cytosol (figure 1*b*) [52,53,80,81]. Similar to extracellular LBP, intracellular GBP1 can recognize either bacterial OMs or LPS micelles and can recruit other GBP family members to the site of detection [82–84]. Caspase-4/11 associates with GBPs and binds to LPS to trigger the activation of the latent enzymatic activity of these proteins, resulting in the caspase-mediated cleavage of the substrate protein gasdermin-D (GSDMD) [82,84–86]. Interestingly, in mice, CD14 also functions to deliver LPS to caspase-11 [87]. After cleavage of GSDMD, the N-terminal subunit oligomerizes at the cell membrane into a large pore [88,89] that can serve as a conduit (i.e. channel) for protein secretion [90]. If these GSDMD pores are not repaired by the cell, membrane rupture may occur by a process known as pyroptosis [91]. Upon pore formation by GSDMD, potassium ions are released into the extracellular space, which leads to the activation of a SMOC known as the NLRP3 inflammasome [92]. Just as the myddosome represents the subcellular site of inflammatory kinases that drive transcription factor activation, the inflammasome is the subcellular site of enzymes that promote the activation and release of IL-1 family cytokines. These inflammasome-associated enzymes are members of the caspase family, most commonly caspase-1 or caspase-8 [93]. The need for caspase-1 to promote IL-1 release is based on the finding that caspase-1 and caspase-4, -5, -11 differ in their abilities to detect LPS and cleave IL-1 [81,92]. Caspase-1 can cleave IL-1*β* [94,95], which is critical to enable its inflammatory activity and transmission through a GSDMD pore [96], but this enzyme cannot bind LPS. Conversely, caspase-4, -5, -11 can bind LPS, but cannot cleave IL-1 family cytokines [52,53]. Thus, inflammasomes serve as a link between the LPS sensory caspases and the caspases that cleave IL-1. Interestingly, whereas the structures and functions of the CD14-MD2-TLR4 network are largely conserved in mammals [97], with the possible exception of bats [98,99], the structure and activities of LPS-binding caspases. The caspase-11 (-4,-5) homologues in carnivores and felines have distinct features from their murine and human counterparts [100,101]. Recent studies established three classes of caspase-4 family members that exist in nature. One class represents LPS receptors that need downstream inflammasomes to process IL-1*β* (e.g. human and mice). One class represents receptors that intrinsically link LPS detection to IL-1*β* cleavage, bypassing the need for inflammasomes and serving as one-protein signalling pathways (felines). The final class represents caspase-1 like proteins that do not bind LPS at all (canines) [102,103].

Lastly, BAI1 functions in concert with TLR4 and LPS-detecting caspases as a PRR that is required to successfully eliminate Gram-negative bacterial infections (figure 1*a*, 14–18) [104]. BAI1 recognition of LPS induces the production of microbicidal reactive oxygen species (ROS) in phagocytes. Originally, BAI1 was discovered because it is abundant in neurons and glial cells and was reported as an angiogenesis inhibitor in a brain tumour model [105]. It is now appreciated that BAI1 is expressed in a wider range of cell types, including macrophages, though at lower levels of expression. Das *et al*. [106] revealed the ability of BAI1 to act as a PRR for LPS. BAI1 is an adhesion-type G-protein-coupled receptor containing an N-terminal extracellular domain that recognizes multiple substrates, including LPS, [104,106] and exposed phosphatidylserine on apoptotic cells [107]. Upon binding to LPS, the intracellular C-terminal domain of BAI1 interacts with the engulfment and cell motility (ELMO) protein and the dedicator of cytokinesis (DOCK180) protein complex, to initiate phagocytosis and NADPH-oxidase-mediated killing of Gram-negative bacteria by ROS in a ras-related C3 botulinum toxin substrate 1 (Rac1)-dependent manner [104]. Unlike the above-described PRRs, BAI1 has been reported to interact with the polysaccharide portion of LPS and not the lipid A subregion [106], suggesting that there are multiple mechanisms of interaction with LPS.

## 4. Lipid A structure and the human-centric view of lipid A detection

The ability of LPS to interact with PRRs is directly related to its structure. The structure of LPS consists of three distinct regions: (i) the lipid A anchor (or subregion), which is hydrophobic, (ii) the core oligosaccharide, which is hydrophilic, and (iii) the O-antigen which is a long chain polysaccharide that is also hydrophilic [44,108,109]. The biosynthesis of each region is tightly regulated on the inner membrane facing the bacteria cell cytosol, beginning with the biosynthesis of the lipid A anchor, followed by the addition of the core oligosaccharide, and ending with the addition of the O-antigen [108]. At the end of this process, LPS is flipped from the cytoplasmic inner membrane to its final destination in the outer leaflet of the OM [110,111]. The lipid A anchor is embedded in an underlying phospholipid bilayer of the OM [32] and has the most conserved structure of the three regions that make up LPS [44,112]. Compared to the lipid A anchor, the hydrophilic regions of LPS are less conserved and typically are not required for bacterial viability in the laboratory setting. The combination of sugars that make up the core and O-antigen of the LPS molecule varies between genera (even species) of bacteria [43,110]. Importantly, the lipid A region of LPS is responsible for the engagement of TLR4 and caspase-11 (-4, -5) [113]. When LPS is stripped of its core and O-antigen, it is still capable of interacting with these mammalian PRRs [16,114–116].

**Figure 2.**
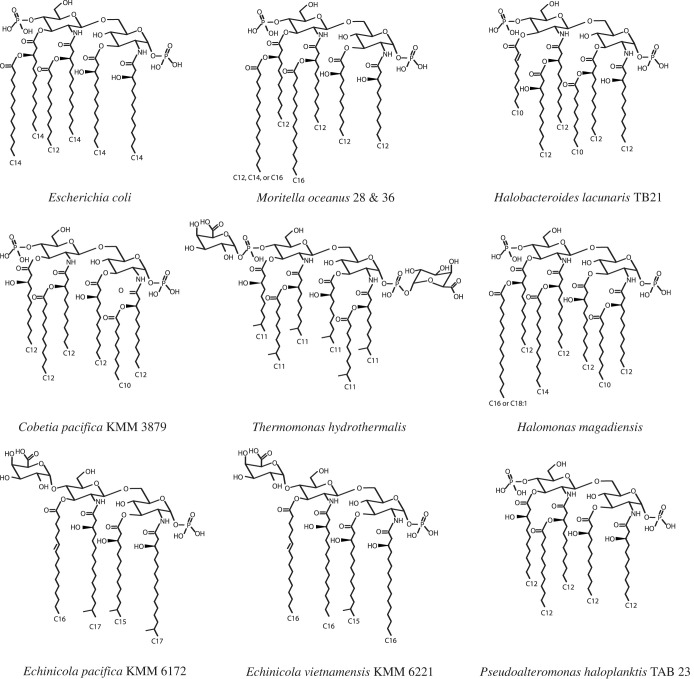
Published structures of lipid A from Gram-negative bacteria cultured from aquatic ecosystems. (*a*) Hexa-acylated lipid A from *E. coli* with acyl chains ranging from 12 to 14 carbons in length [108]. Compared to *E.coli* lipid A, (*b*) hexa-acylated lipid A from *M. oceanus* 28 and 36 contains longer secondary acyl chains with 16 carbons [117], (*c*) hexa-acylated lipid A from *H. lacunaris* TB21 contains shorter primary and secondary acyl chains with 10 carbons [118], (*d*) hexa-acylated lipid A from *C. pacifica* KMM 3879 contains a shorter acyl chain with 10 carbons [119], (*e*) hexa-acylated lipid A from *T. hydrothermalisis* contains only shorter, odd-length primary and secondary acyl chains with 11 carbons as well as D-GalA residues attached to the phosphate groups of the di-glucosamine backbone [120], (*f*) hepta-acylated lipid A from *H. magadiensis* contains shorter (10 carbons) and longer acyl chains (16 or 18 carbons) [121], (*g*) tetra-acylated lipid A from *E. pacifica* KMM 6172 T contains longer primary acyl chains ranging from 15 to 17 carbons as well as a D-GalA residue attached to the di-glucosamine backbone [122], (*h*) tetra-acylated lipid A from *E. vietnamensis* KMM 6221 contains longer primary chains with 15 or 16 carbons as well as a D-GalA residue attached to the di-glucosamine backbone [122] and (*i*) penta-acylated lipid A from *P. haloplanktis* TAB 23 contains only even-length acyl chains with 12 carbons [123].

The structure of *E. coli* lipid A is largely conserved among aerobic and enteric Gram-negative bacteria (figure 2*a*) [44,124]. This lipid A is composed of a bis-phosphorylated *β*(1→6)-linked D-glucosamine disaccharide backbone that is hexa-acylated with four primary acyl chains and two secondary acyl chains. Primary acyl chains are linked to the disaccharide backbone via ester and/or amide bonds, and typically, the secondary acyl chains extend asymmetrically from the primary acyl chains [18,108,109]. The saturated acyl chains of lipid A are aliphatic in nature, and it is this property that creates the low permeability environment for hydrophobic solutes [32,36]. *E. coli* lipid A has acyl chains ranging from 12 to 14 carbons in length [108,109]. Experiments measuring bacteria membrane permeability after altering the number of acyl chains present in *E. coli* lipid A resulted in increased permeability when the number of acyl chains was experimentally decreased [125], illustrating that hexa-acylation is required for optimal bacterial membrane function and integrity in *E. coli*. Acyl chain number is likely to be species-specific to optimize permeability for each taxa dependent on the environmental setting [44,126–128].

Central to the ideas of pattern recognition, as it relates here to LPS-PRR biology, is that multi-cellular organisms detect conserved cell wall structures as their primary signal of microbial encounter. This strategy enables a small set of LPS-binding proteins to detect the conserved cell wall structure of any bacterium we would ever encounter. As such, the idea of pattern recognition predicts that all bacteria should be recognizable by PRRs, except those bacteria that are host-adapted and have evolved PAMPs capable of immune evasion [113,124,128,129]. Based on the human-centric work of most research in this area, the tenets of the pattern recognition concept have been most thoroughly explored using bacteria and LPS that have the potential to interact with terrestrial animals. The most laboriously studied examples include studies of *E. coli* and other pathogens and commensals of humans [127,130–137]. Based on this work, bis-phosphorylated, hexa-acylated lipid A (with specific carbon chain number) is considered the ideal structure in Gram-negative bacteria, and this structure is near-universally detected by mammalian PRRs. Alteration of this structure to evade PRRs only occurs when evolutionary pressures of bacterial detection by the host are applied (as in the case of virulent pathogens or beneficial commensals). However, it is important to note that most multi-cellular organisms are not terrestrial, and most bacteria live in environments that are not conducive to human life and may never interact with humans. In the next section, we discuss recent studies to determine the role of the lipid A subregion of LPS from bacteria that occupy diverse ecological niches to serve as PAMPs for mammalian PRRs.

## 5. Lessons from the environment: Gram-negative bacteria

To determine whether lipid A has a globally conserved structure and elicits a global pattern of conserved response, Gram-negative bacteria have been cultured from diverse, and in some cases extreme, environments to determine the lipid A structure of LPS and its effect on the mammalian innate immune system (figure 2*b–i*). A recent study posed the question of whether PRRs of mammals could detect LPS from bacteria from an environment largely uninhabited by mammals: the deep sea [117]. The deep sea harbours no resident mammals, and there are a limited number of mammalian species that access it via sporadic diving [138]. The deep sea is also known to host distinct populations of Gram-negative bacteria, different from shallow water and terrestrial environments [139,140]. Deep-sea samples collected from the equatorial Pacific were found to be rich in a culturable genus of bacteria, *Moritella spp.*, that is not found in shallow water samples from the same region [117]. Therefore, *Moritella spp*. offered a model to test the universality of the concepts of pattern recognition.

Using a newly discovered species of deep-sea Gram-negative bacteria, *Moritella oceanus*, Gauthier *et al*. demonstrated that these bacteria have an OM composed of lipid A that was not detected by CD14, TLR4 or caspase-11. This finding was notable, as subsequent analysis revealed that *M. oceanus* LPS contained *E. coli*-like lipid A structures that were hexa-acylated, bis-phosphorylated, and displayed no other backbone modifications. The immunological silence of *M. oceanus* lipid A was associated with an increased level of C16 secondary acyl chains (figure 2*b*). The high levels of C16 could explain the inability of *M. oceanus* lipid A to be detected by the mammalian PRRs tested [117]. Previous findings support that C16 may prevent interaction of lipid A with mammalian MD2, and hence, TLR4 engagement [141]. However, why C16 chains would interfere with CD14 interactions and caspase-11 interactions remains unknown. Thus, the interaction between acyl chain length and PAMP recognition is a ripe area for future investigation.

It is appreciated that the conditions of an environment directly influence bacterial membrane structure; specifically, the OM must adapt for optimal function and fitness of the bacteria [142–145]. Extreme environments such as the deep-sea thus present an opportunity to test the hypothesis that environmental drivers of bacterial fitness may be vastly different from a terrestrial or mammalian-host enteric environment and therefore may not be compatible with mammalian PRR systems. A recent study tested this hypothesis by screening the ability of 44 strains of live *Moritella sp.* to engage CD14 and TLR4 in mouse macrophages. It was found that 80% of the strains tested were unable to engage with CD14 and/or TLR4, as compared to live *E. coli*, even though all strains were predicted to contain a bis-phosphorylated, hexa-acylated lipid A [117]. It is almost certain that deep-sea bacteria did not evolve to evade mammalian PRRs and do not gain any fitness benefit from doing so. Instead, this environment should have a different set of evolutionary drivers and constraints, independent of mammalian interactions, such that there is no evolutionary pressure for LPS to be immune evasive, stimulatory or silent in mammals. This idea therefore implies that any immuno-stimulatory or immuno-evasive response of mammalian cells to deep-sea LPS is accidental rather than selectively driven. Importantly, this raises the question as to the frequency at which this phenomenon happens when bacteria are isolated from other extreme environments. Further tests of the pattern recognition model with lipid A from diverse ecosystems remain an important topic for future investigation. Indeed, a solid experimental foundation for such ecosystem-based tests of the universality of pattern recognition already exists.

For example, lipid A structures have been isolated from the Gram-negative bacteria *Halobacteroides lacunaris* TB21, which was cultured from a deep-sea hypersaline anoxic brine in the Eastern Mediterranean Sea [118], and also from *Cobetia pacifica* KMM 3879 cultured from shallow water sediment collected in the Sea of Japan [119] (figure 2*c,d*). Interestingly, both of these bacteria produced hexa-acylated lipid A species with C10 and C12 acyl chains, and the LPS from both species behaved similarly in murine macrophages and human cells. In these cells, the marine lipid A structures were poor inducers of NF-κB activation and competed with *E. coli* LPS for receptor binding. These findings suggested that these lipid A structures bind but do not signal. There is a growing list of bacteria with a variety of acyl chains of varying lengths from aquatic environments, and interestingly, many of these provoke a reduced innate immune response compared to *E. coli*, suggesting the potential for pseudo-silent or highly dampened immune activation. *Thermomonas hydrothermalisis* was first cultured from a hot spring in Gemil, Portugal and produces a hexa-acylated lipid A with C11 acyl chains and galacturonic acid (D-GalA) residues attached to the phosphate groups of the di-glucosamine backbone (figure 2*e*). In murine macrophages and human cells, LPS from *T. hydrothermalisis* failed to induce innate immune signalling as measured by NF-κB activation and cytokine release compared to *E. coli* LPS [120]. *Halomonas magadiensis* is a halophilic Gram-negative bacteria that expresses a hepta-acylated lipid A with even-length acyl chains (C10, C12, C14, C16 and/or C18) and is similarly incapable of activating NF-κB in HEK293 cells and human THP-1 cells (figure 2*f*) [121]. *Echinicola pacifica* KMM 6172 T and *Echinicola vietnamensis* KMM 6221 were isolated from animals inhabiting marine environments: a *Strongylocentrotus intermedius* urchin from the Sea of Japan and mussels from a farm on Nha Trang Bay in the Sea of China, respectively [122]. Both produce a monophosphorylated, tetra-acylated lipid A characterized by odd and even-length acyl chains (C15, C16 and/or C17) as well as a D-GalA residue on the glucosamine sugar that is not phosphorylated (figure 2*g*,*h*). The lipid A from both species induced significantly less NF-κB activation and IL-8 release compared to *Salmonella enterica* serovar Typhimurium. *Pseudoalteromonas haloplanktis* TAB 23 was isolated from shallow water in the Antarctic Sea and was found to produce a penta-acylated lipid A with C12 length acyl chains that was incapable of inducing the production of tumour necrosis factor *α* (TNF*α*) in human THP-1 cells (figure 2*i*) [123].

Collectively, these publications suggest that there are some Gram-negative bacteria that may have an increased occurrence of producing lipid A that is antagonistic or is functionally silent (pseudo-silent) to mammalian PRRs. In the above examples, lipid A chain length and number are directly implicated in the biological activity of immune interactions, but this coupling of structure and function is relatively rare in the environmental microbiology literature. All of the above examples have been isolated from aquatic environments, but whether similar characteristics have evolved in other environments remains unexplored. It may be hypothesized that the wider the difference in physical environment between bacteria and a mammalian host, the greater the chances of accidental mammalian PRR evasion, pseudo-silence or silence. It is unclear whether this same hypothesis should be applied to PRRs produced by non-mammals, which represent the vast majority of multi-cellular life forms.

## 6. Lessons from the environment: eukaryotes

Across the tree of life, lipid A (or LPS) has been used to study the activation of non-mammalian innate immunity. In experimental settings, the most common approach is to treat cells or inject animals or plants with purified LPS or lipid A from *E. coli* [113]. While this is a logical experimental starting point from a mammal-centric perspective, mammals represent a small fraction of all multi-cellular life. Mammals represent less than 1% of all animals. Invertebrates account for approximately 95% of all animals on Earth, and lower vertebrates (amphibians, fish and birds) comprise a bulk of the remaining 5%. When plants are included in these figures; plants account for about 20% of all species of multi-cellular life compared to animals [146]. The spectrum of non-mammalian organisms that detect LPS is largely undefined. Even in some common model organisms, the ability to detect lipid A is unknown or unclear. In general, evidence to date underscores that the use of lipid A as a PAMP across taxa is not conserved; we will review lipid A as a PAMP (or not) in common model organisms below.

In some invertebrates, lipid A is a PAMP, similar to what has been observed in mammals. Lipid A detection in the horseshoe crab (*Limulus polyphemus*) by the PRR Factor C is well established, following a molecularly defined activation pathway [147–151]. In granular hemocytes, the immune cells of horseshoe crabs, the serine protease Factor C is expressed on the plasma membrane and remains inactive until it recognizes lipid A, upon which time it cleaves itself into its active form [147,152]. Active Factor C cleaves the zymogen Factor B, which subsequently activates the pro-clotting enzyme that is key to the conversion of coagulogen to coagulin and the antimicrobial clotting response to remove any potentially harmful bacteria [149,153–156]. As a part of this response, it has been proposed that coagulin may serve as a ligand to a TLR identified in horseshoe crabs to induce downstream NF-κB signalling [157–159]. Factor C is sensitive to picogram quantities of lipid A, and for this reason—coupled with the high toxicity of lipid A to humans—*L. polyphemus* are harvested to isolate Factor C from their blood as a highly sensitive assay to detect endotoxin (lipid A) contamination in medical and pharmaceutical industries [160–162]. Factor C does share an important and surprising commonality with mammalian LPS receptors: it could not detect lipid A from deep-sea *M. oceanus,* suggesting that long-held assumptions of the universality of lipid A detection by Factor C are incomplete [117].

By contrast, there are some model systems that do not have any mechanism to detect LPS. For example, the model organism *Drosophila melanogaster* does not express any lipid A detecting PRRs and therefore does not elicit a transcriptional response to any LPS or lipid A [163,164]. In *D. melanogaster*, the detection of Gram-negative bacteria requires the immune deficiency (IMD) pathway and recognition of the PAMP peptidoglycan (PGN) [163,164]. Previous data that purportedly show LPS activating the innate immune system of *D. melanogaster* are a result of PGN contamination of LPS preparations [165,166], which was later clarified through a series of definitive experiments. Early (pre-2000) experiments suggesting LPS detection all require repeated verification, because the protocol for successfully purifying LPS was not established until 2000 [167]. Using robust protocols, Leulier *et al*. used purified LPS and PGN derived from the cell walls of Gram-negative and Gram-positive bacteria to illustrate that only PGN, but not LPS, could stimulate innate immune responses in *D. melanogaster* [164]. Similarly, experiments performed by Kaneko *et al*. with synthetic lipid A, which is free of contaminants, underscored that LPS does not activate the IMD pathway, but PGN from Gram-negative bacteria does [163].

The nematode model organism, *Caenorhabditis elegans*, also may not detect lipid A. Instead, there is evidence that other portions of the LPS molecule (O-antigen and core) mediate innate immune signalling. Aballay *et al*. observed *Salmonella enterica* with mutations in core and O-antigen biosynthesis enzymes could not mount a productive immune response in *C. elegans*, despite the fact that lipid A remained intact (unchanged) [168]. *S. enterica* with intact LPS induced an innate immune defence pathway known as the programmed cell death (PCD) response in nematodes. When the authors of this study purified LPS from *S. enterica*, however, they could not induce the PCD response observed with live infections. It is possible that LPS is not a specific PAMP in nematodes, but that intact LPS is required for non-specific cell adhesion in the context of infection. To date, no PRR that recognizes LPS has been identified in *C. elegans*. The genome of these organisms, however, does contain one TLR orthologue, a TIR domain-containing gene, and nine LBP orthologues [169,170]. Unlike in mammals and *D. melanogaster*, the *C. elegans* TLR does not participate in the innate immune responses to most microbial stimuli [171], however, the TIR domain-containing protein (TIR-1) is critical to the innate immune response against mutliple Gram-negative pathogens [171–173]. Whether the LBP orthologues in *C. elegans* have a role in host defence is unknown, however, one gene (F44A2.3) is upregulated in response to the Gram-positive bacteria, *Enterococcus faecalis*, and the Gram-negative bacteria, *Photorhabdus luminescens* [174].

Until recently, it was thought that the model mustard plant *Arabidopsis thaliana* recognizes lipid A from *Pseudomonas spp*. and *Xanthomonas campestris*, but not *E. coli*, via the membrane-bound PRR, lipooligosaccharide-specific reduced elicitation (LORE) [175]. However, a recent study refuted this idea with the findings that LPS and lipid A from commercial and laboratory-derived preparations contain free medium-chain 3-hydroxy fatty acids, essentially confounding the conclusions of studies that were previously assumed to have LPS- and lipid A-only preps. Kutschera *et al*. [176] found that the removal of these free fatty acids from LPS and lipid A preparations rendered the LPS and lipid A silent to LORE-mediated innate immune detection, thereby suggesting that the free fatty acids were the detection trigger. The authors concluded that LORE binds most strongly to medium-chain 3-hydroxy fatty acids with 10 carbons, and further hypothesized that these fatty acids are a product of separate cellular processes in bacteria and are not always directly attached to lipid A naturally. In *Pseudomonas spp.*, however, medium-chain 3-hydroxy fatty acids with 10 carbons are a derivative of lipid A that may functionally behave as an indirect LPS detection trigger [177]. Whether or not the fatty acids are released in the context of an innocuous encounter or in direct response to *Pseudomonas* infection in *A. thaliana* remains unknown.

Although extracellular lipid A pattern recognition clearly occurs in marine environments (*L. polyphemus*), it is thought that large classes of lower marine vertebrates are functionally blind to extracellular lipid A. For example, fish are extremely resistant to LPS-induced septicemia and can survive injections of upwards of 200 µg kg^−1^ of purified LPS [178–180]. Initially, these data stimulated the theory that there may be no PRR in teleost fishes (Osteichthyes) to detect lipid A. Indeed, orthologues of TLR4 and their accessory proteins (MD2 and CD14) are virtually all absent from the sequenced genomes of teleost fish [178,181–184]. Sequencing of the elephant shark genome (Chondrichthyes) revealed that the TLR4 gene contained numerous stop codons which would undoubtedly render it non-functional, prompting the hypothesis that Chondrichthyes also do not detect LPS, at least extracellularly [185]. Even though high quantities of LPS are not lethal to fish, in clear contrast with the mammalian response, there are numerous reports of fish mounting a transcriptional response to LPS [186–191]. These reports, however, have used doses of LPS (10–500 µg ml^−1^) that are 100–5000 times higher than used in mammals. It is only at these high doses that a transcriptional response is detected, and even still, fish are resistant to LPS-induced septicemia. As such, extracellular LPS detection at an environmentally relevant dose may not naturally occur in fishes.

An interesting debate surrounds the common model freshwater fish *Danio rerio*, which expresses two paralogues of TLR4 (TLR4ba and TLR4bb) [192–194] and a putative orthologue of MD2 (LY96) [195]. However, these paralogues do not activate NF-κB upon stimulation with LPS *in vivo* or when transfected into a 293 T reporter cell line alone or in tandem with human CD14 and MD2 [193,194]. Furthermore, co-expression of TLR4ba and TLR4bb with *D. rerio* LY96 did not activate NF-κB. Interestingly, when TLR4bb was overexpressed with LY96 and human CD14, Loes *et al*. observed the activation of this pathway. However, this surprising finding still suggests that paralogues or orthologues to mammalian TLR4, and its accessory proteins are not lipid A sensing PRRs in *D. rerio* [195], since it was only with a mammalian accessory protein that the activation of NF-κB could be achieved. No other fish to date has been shown to possess extracellular PRRs that detect lipid A [189,192], though this requires further investigation. Given the above experiments, *D. rerio*-specific TLR4ba or TLR4bb are probably not responsible for an innate immune response to LPS, but notably, transcriptional responses and/or animal death upon prolonged exposure to high concentrations of LPS were observed [195]. As such, we hypothesize that intracellular lipid A pattern recognition may instead be responsible for the phenotypes reported. To this point, there has been direct evidence that *D. rerio* expresses a functional orthologue of caspase-4/11 that senses LPS in the cytosol [196,197]. Notably, published genomes of teleost fishes do contain putative orthologues of intracellular lipid A detecting caspases; but there are no other lipid A detecting caspases in fish that have been functionally confirmed. As such, mechanisms of potential intracellular LPS detection require further study in aquatic systems.

Given the complexity of potential extracellular and intracellular LPS and lipid A detection pathways across eukaryotic domains, a clear understanding of where and when lipid A recognition has been gained and lost throughout evolutionary history remains an unsolved puzzle. It may not be as simple as saying certain classes of eukaryotes have PRRs that recognize lipid A while others do not. For example, insects (excepting the model organism *D. melanogaster*) do display evidence of innate immunity to lipid A, although no PRRs have been identified to date [198], and statements like these are possible for many multi-cellular organisms. If one examines the literature for a species’ response to LPS or lipid A, there is undoubtedly at least one publication describing the engagement of an inflammatory innate immune pathway, but no identification of a PRR; hence highlighting how rich the field of lipid A pattern recognition is for discovery. Moreover, little is known about the lipid A structures required for optimal innate immune recognition in non-mammalian eukaryotes. It is possible that no PRRs have been identified in some of the model species described above because the optimal lipid A structure is unknown. Given the diversity of lipid A structures from bacteria cultured from diverse environments, it is possible that lipid A from an organism's endogenous environment could reveal new PRRs for LPS. The recent evidence that even small changes of 2 carbons in lipid A chain length can alter the ability of PRRs to detect LPS in mammals [117–120,141] suggests that significant structure-function considerations must be made when such investigations are undertaken.

## 7. Discussion: global pathogen-associated molecular pattern detection in eukaryotes

From this review, three main conclusions are offered: (i) LPS is a common PAMP in terrestrial mammals, with multiple PRRs operating to induce complementary yet distinct host responses upon encountering this microbial product; (ii) the focus of PRRs on LPS as a means of bacterial detection is most likely to be a mammal-specific trait that is best suited to detect bacteria that co-inhabit the same terrestrial ecosystem; and (iii) lipid A is not a PAMP in all multi-cellular organisms. These conclusions raise questions about the recognition of other PAMPs. Are they conserved throughout nature, in terms of their mechanisms of detection by multi-cellular hosts and are global detection mechanisms common to broad groups of eukaryotes? The answer for lipid A is clearly no, but there may be other PAMPs that are more global and conserved. Thus, the real question may instead be, why is lipid A detection not used by all multi-cellular organisms to detect bacteria? Considerations of the mechanisms of PAMP detection, and the corresponding risks and benefits of any given pathway, are key to understanding the potential exploitative routes of infection across the evolutionary spectrum. To explore these questions, we first examine the universality premise of PAMP detection in non-lipid A molecules.

Microbial nucleic acid sensing PRRs are an obvious candidate for universal PAMPs, as all microorganisms possess nucleic acids (genetic material) foreign to multi-cellular organisms. Mammals recognize foreign nucleic acids through PRRs localized to endosomes, the nucleus and the cytosol. TLRs, RIG-I-like receptors (RLRs), the DICER family of proteins (DICER) and individual PRRs AIM2, IFI16, cGAS and STING recognize microbial nucleic acids in mammals. Substrates recognized by intracellular nucleic acid sensing PRRs include double-stranded (ds) RNA (TLR3, RLRs and DICER), single-stranded (ss) RNA (TLR7/8), CpG DNA (TLR9), 23 s rRNA (TLR13), dsDNA (AIM2, IFI16 and cGAS) and cyclic dinucleotides (STING) [199,200]. While there is a multitude of nucleic acid substrates, detection by the corresponding PRRs can occur at different locations within the cell. For example, human and murine PRRs that recognize nucleic acids of microbes are localized exclusively to the intracellular space of the cell [201,202]. TLRs localized to the endosome detect nucleic acids from microbes surveyed from the extracellular space, while cytosolic and nuclear PRRs detect nucleic acids generated by intracellular pathogens as part of their life cycle [201,203]. Many PRRs that recognize nucleic acid PAMPs in mammals induce the production of interferon, with the exception of the pyroptosis-inducing receptor AIM2 [204,205] and the DICER family of proteins [200]. Mammalian DICER proteins possess the ability to cleave dsRNA into small RNAs [206–208]; however, interferon-mediated responses can suppress DICER signalling and account for the majority of the innate immune response induced following the detection of microbial nucleic acids [203,209,210]. The interplay between DICER and interferon signalling is actively being defined and is multi-layered [211]. Importantly, interferon-mediated innate immunity is conserved in vertebrates including teleost fishes as well as Chondrichthyes. Teleost fishes use orthologous PRRs to mammalian TLRs, RLRs, cGAS-STING and probably DICER; however, AIM2 and IFI16 are absent in fish genomes [212–216]. Interestingly, fish have a unique TLR, TLR22, that is localized to the plasma membrane and recognizes dsRNA [217]. Thus, even if nucleic acids are globally detected, the pathways for detection may be varied across taxa.

In invertebrates and plants, interferon is absent, and the recognition of microbial nucleic acids is accomplished by multiple classes of PRRs independent of interferon [218,219]. *D. melanogaster* innate immune signalling pathways responsible for the detection of foreign nucleic acids include the Toll pathway [220], the IMD pathway [221], the cGAS-STING pathway [222–224] and the RNA interference (RNAi) pathway [225–227]. While the role of the Toll and IMD pathways in these contexts is obscure, it is established that cGAS-STING and DICER2 bind microbial nucleic acids*.* Intracellular recognition of dsDNA by *D. melanogaster* cGAS-STING induces NF-κB and is required to restrict viral infection [222,224,228]. DICER2 binds dsRNA and catalyses its conversion to short interfering RNAs (siRNAs) that are subsequently loaded onto downstream host proteins to be degraded [225,226,229,230]. Notably, only DICER orthologues are present in the genomes of *A. thaliana* and *C. elegans*, while orthologues to cGAS and STING are absent [231,232]. *D. melanogaster* and *A. thaliana* express multiple DICER proteins and use specific DICER proteins (DICER2 in *D. melanogaster* and DICER1–4 in *A. thaliana*) directly as PRRs that bind foreign nucleic acids [233–235]. By contrast, the *C. elegans* genome has one DICER protein that potentially partners with multiple PRRs to exert an innate immune response [232,236]. *C. elegans* expresses orthologues of RLRs (RDE4 and DRH1) that are required for RNAi and function as PRRs for nucleic acids in concert with DICER [237,238]. Apart from RNAi, *A. thaliana* can induce two other innate immune defence pathways upon recognition of foreign nucleic acids. In plants, PAMP-triggered immunity (PTI) and nuclear shuttle protein-interacting kinase 1 (NIK1)-mediated immunity are initiated following recognition of viral nucleic acids, RNA and DNA, respectively [239]. The leucine-rich repeats (LRR) receptor-like kinases in *A. thaliana*, somatic embryogenesis receptor kinase 1 (SERK1) and the brassinosteroid-insensitive 1 (BRI1)-associated receptor kinase 1 (BAK1), are coreceptors for unknown PRRs that detect RNA viruses and induce PTI. Similarly, the PRR that functions as a coreceptor with NIK-1 is unknown [240]. Unlike the above-described model plant and invertebrates, the PRRs and signalling pathways that respond to microbial nucleic acids in *L. polyphemus* have not been identified. Interestingly, DICER orthologues are present in the terrestrial arachnid species, *Tetranychus urticae*, and RNAi molecular machinery is functional [241]. *L. polyphemus* does encode genes orthologous to those present in the *D. melanogaster* Toll and IMD signalling pathways; however, whether these pathways are important for an innate immune response to nucleic acids in these animals remains unknown [242]. Therefore, apart from *L. polyphemus,* the recognition of microbial-derived nucleic acids is established in plants and invertebrates even in the absence of interferon.

PRRs that recognize the bacterial cell wall component, PGN, are a second plausible candidate for a universally detected PAMP. PGNs are peptide-linked polysaccharides in the bacterial cell wall with specific features that are critical to the structure and function of both Gram-negative and Gram-positive bacteria [243]. The sugar backbone of PGN is primarily composed of two disaccharides, N-acetylmuramic acid (NAM) and N-acetylglucosamine (NAG). The linked peptides in Gram-negative bacteria are often distinguished by γ-d-glutamyl-meso-diaminopimelic acid (iE-DAP) [244], while Gram-positive bacteria contain an L-Lysine residue and sometimes iE-DAP [245]. Both classes of bacteria possess muramyl dipeptide (MDP), the minimal region of PGN required for interacting with some mammalian PRRs [246]. Apart from these conserved features, the number of sugars comprising the PGN backbone, and their modifications, can vary greatly between different bacterial species [243,245,247,248]. The best-characterized PRRs that detect Gram-negative PGN in mammals are the nucleotide-binding oligomerization domain (NOD) proteins, NOD1 and NOD2, which are localized to the cytosol and detect iE-DAP or MDP, respectively [249–251]. NOD2 can also recognize Gram-positive bacteria via MDP. In addition to these receptors, the following PRRs have been identified in mammals that recognize conserved components of PGN: the nucleotide-binding domain and LRR-containing (NLR) family of receptor members, NLRP1 [252,253] and NLRP3 [254,255], which are localized to the cytosol as well as PGNs recognition proteins 1–4 (PGRP1–PGRP4), which are secreted [256,257]. Unlike NOD1, NOD2, NLRP1 and NLRP3, which induce proinflammatory transcriptional responses in mammals following the recognition of PGN, PGRP1, 3 and 4 kill bacteria bound via PGN by disrupting the function of the bacterial cell membrane [256,258–261]. Human PGRP2, however, has amidase activity and hydrolyses PGN from the bacterial cell wall, possibly for subsequent detection by NOD receptors [262,263]. Similar to what was observed for nucleic acids, the model organisms evaluated encode PGN-sensing PRRs, lending initial support for broad recognition of PGN molecules.

Further examining PGN recognition across eukaryotic taxa, it has been shown that genomes of teleost fishes contain orthologues to mammalian PRRs NOD1, NOD2 and PGRP that recognize PGN; however, only PGRPs of zebrafish have been functionally verified to bind PGN of Gram-negative and Gram-positive bacteria [216,264–266]. Apart from mammals, PGN-sensing PRRs are most well-characterized in *D. melanogaster* which binds PGNs via long or short PGRPs [163,164,267–273]. PGRP-LC and PGRP-LE are long receptors localized to the extracellular space, the plasma membrane, or the cytosol that bind Gram-negative bacteria through PGN-containing iE-DAP and activate the IMD pathway [163,164,274]. PGRP-SA and PGRP-SD are short receptors that survey the extracellular space and induce the Toll pathway following the recognition of Gram-positive bacteria with PGN-containing L-lysine residues [275,276]. In total, the genome of *D. melanogaster* encodes 13 PGRPs, some of which bind to PGN and have functional consequences independent of the Toll and IMD pathways [268,277]. PGRPs are conserved in the model invertebrate *L. polyphemus* [242]; however, the existence of a PGRP orthologue in *C. elegans* is debatable [256,278]. Interestingly, the Toll and IMD pathways are present in *L. polyphemus*, suggesting that these pathways may function downstream of PGRPs, but this remains to be tested. On the other hand, should an orthologue of PGRP function as a PRR in *C. elegans,* it does not employ the Toll or IMD signalling pathways, as these pathways are absent in nematodes [171,279]. In contrast with invertebrates, fish and mammals, *A. thaliana* does not employ PGRPs as PGN-sensing PRRs. Instead, plasma membrane-localized lysin-motif (LYM) proteins, LYM1 and LYM3, bind PGN from both Gram-negative and Gram-positive bacteria and induce PTI [280,281]. Therefore, all the model organisms discussed encode PGN-sensing PRRs, but only the PRRs of *D. rerio*, *D. melanogaster* and *A. thaliana* have been functionally verified apart from mammals.

**Figure 3.**
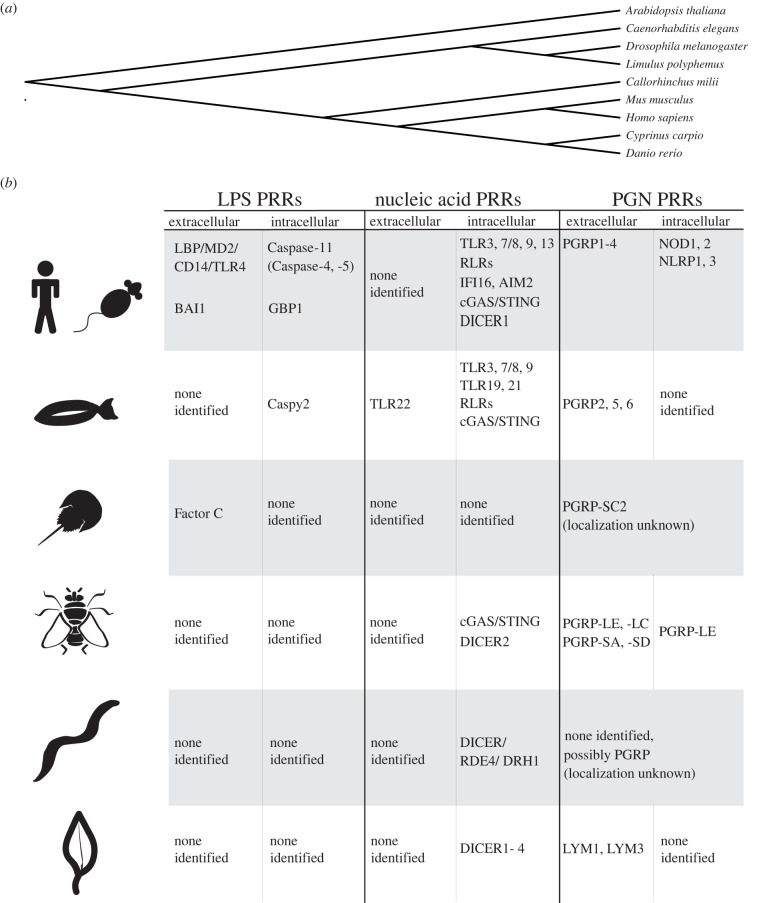
PRRs are identified in mammals and other commonly used model organisms. (*a*) Evolutionary tree [282] of common model organisms is discussed in the text. (*b*) Table of PRRs is identified in common model organisms that recognize LPS, nucleic acid and PGN PAMPs.

Innate immune recognition of microbial nucleic acids and PGN is likely conserved in plants, invertebrates (*D. melanogaster, C. elegans* and *L. polyphemus*), teleost fishes and mammals through overlapping and unique classes of PRRs. This is in contrast with the lipid A subregion of LPS, a PAMP that may not be recognized by select invertebrates and plants (*D. melanogaster, C. elegans* and *A. thaliana*), as well as possibly teleost fishes (and Chondrichthyes) (figure 3). One hypothesis as to why nucleic acid sensing and PGN-sensing PRRs are more ubiquitous is that these PAMPs are common to multiple classes of microorganisms. Only Gram-negative bacteria possess LPS, whereas nucleic acids are common to all classes of microorganisms and PGN is present in the cell walls of both Gram-negative and Gram-positive bacteria. When PAMPs are common to multiple classes of microorganisms, there may be a stronger evolutionary advantage for PRRs to recognize these PAMPs in a greater range of multi-cellular organisms.

## 8. The future of understanding lipopolysaccharide lipid A as a pathogen-associated molecular pattern in innate immunity

The near-universal detection of nucleic acids and PGN in the organisms discussed raises the question as to why the lipid A subregion of LPS did not evolve to be a globally detected PAMP. We propose two new hypotheses here that could be tested to address this question.

Hypothesis 1: *The density of lipid A in the environment influences LPS-sensing PRR evolution: high density Lipid A will desensitize PAMP-response, and thus select against LPS-sensing PRR evolution*.

The concentration of Gram-negative bacteria in the environment may have influenced the evolution of LPS-sensing PRRs. For example, in marine environments, the concentration of bacteria in shallow water (less than 200 m) on average is 1 × 10^6^ ml^–1^ [140,283], whereas in the air, it is 1 × 10^3^ ml^–1^ [284]. Perhaps, because LPS is located on the cell surface of Gram-negative bacteria coupled with the high concentration of bacteria in shallow ocean water, certain marine organisms evolved not to detect the lipid A subregion of LPS extracellularly, or altogether, because it is too prevalent to effectively work as a detection signal. This hypothesis may, however, be localized to specific environments and/or be heavily influenced by life-history traits. For example, *L. polyphemus* serves as an example of a marine organism that recognizes lipid A, but the concentrations of lipid A in the natural environment at the time of *L. polyphemus* evolution (445 Ma) are unknown [285]. Further, deep-sea invertebrates occupy a habitat with comparable concentrations of bacteria present in the air of terrestrial environments [286], but there are as of yet no data on organismal response to lipid A in this extremely primitive and ancient deep-sea environment.

Hypothesis 2: *Animals that feed on Gram-negative bacteria may have evolved to not recognize lipid A as a PAMP. Predation of Gram-negative bacteria may have selected against PRR-detection to enable food consumption without immunostimulation*.

Some invertebrates consume bacteria as a food source, such as *D. melanogaster* and *C. elegans*; interestingly, these organisms do not use lipid A as a PAMP. In *C. elegans*, only intact LPS can induce an innate immune response, suggesting that only whole bacteria may activate innate immunity; any bacteria in the process of whole or partial consumption would have a disrupted LPS, presumably rendering it undetectable. In *D. melanogaster*, no recognition of any region of LPS occurs; instead, Gram-negative bacteria are detected via PGN and/or nucleic acids. There are many taxa that consume microbes as a food source (e.g. foraminifera [287], copepods [288], rotifers [289], amoebas [290], mites [291] and gastropods [292]), but the PRR detection pathways for these organisms has not yet been investigated except for the social amoeba, *Dictyostelium discoideum*, which is known to express one TIR domain-containing protein that participates in microbial recognition [293]. A cursory look at the genomes of foraminifera and *Dictyostelium discoideum* suggest that TLRs are not present, while orthologues to TLRs are present in copepods, rotifers, mites and gastropods, lending anecdotal justification/incentive for investigating this hypothesis further. Large meta sweeps of rapidly emerging genomic information can help to validate or eliminate this hypothesis, but this work has yet to be undertaken. In other systems, the detection of ‘friend from foe from food’ has been well examined and is critical for organisms to activate the appropriate response [294–298], and the inability to distinguish between these categories has consequences [299]. Microbial predators include some of the earliest-evolved multi-cellular taxa on earth, and so it is possible that they have overly simplistic recognition systems where they cannot distinguish between categories of non-self cells. Alternatively, their long history may have instead enabled selection for complex recognition mechanisms to distinguish between categories of non-self cells. This question remains unanswered but opens new lines of inquiry into the origins of innate immune systems.

## 9. Concluding thoughts

There has been much work on the mechanisms of LPS detection, and the conservation of response across model organism taxa. However, the field is now at the point of needing to understand the evolution of different LPS structures and the coevolutionary response (or lack thereof) in PRRs. Evolutionary pressures unique to an environment may result in some PAMPs, (e.g. nucleic acids and PGN), being more universally detected than lipid A by PRRs of multi-cellular organisms. Likewise, these pressures may also contribute to why PAMPs from distinct ecosystems are accidentally silent or antagonistic to their cognate PRRs in mammals. Whether or not nucleic acids and PGN from innocuous microbes can be accidentally silent to their respective PRRs in derived and basal eukaryotes akin to lipid A in mammals remains unknown and is a ripe area for exploration. A shift in thinking from the ‘what’ to the ‘why’ of PAMP structure and PRR response will undoubtedly help to discover new structures and pathways, but it will also help us to understand the origins of innate immunity and find new PAMP variations of therapeutic interest. The development of novel tools and assays, and their affordability, has created the opportunity to enable rapid insight in natural systems beyond the classic model organisms. Examination in extreme environments and across taxonomic breadth of both microbes and hosts will certainly catalyse transformational advance.

## Data Availability

This article has no additional data.
